# Adenovirus Type 4 Respiratory Infections among Civilian Adults, Northeastern United States, 2011–2015

**DOI:** 10.3201/eid2407.180137

**Published:** 2018-07

**Authors:** Breda L. Lynch, Jonathan Dean, Deirdre Brady, Cillian De Gascun

**Affiliations:** Mater Misericordiae University Hospital, Dublin, Ireland (B.L. Lynch, D. Brady);; National Virus Reference Laboratory, Dublin (J. Dean, C. De Gascun)

**Keywords:** human adenovirus B7, adenovirus type 4, adenovirus, viruses, respiratory infections, cluster, community onset, disease severity, Ireland, United States

**To the Editor:** We read with interest the article by Kajon et al. (*1*), which highlighted that human adenovirus type 4 might be an underrecognized cause of acute respiratory disease (ARD) outside military settings. We report that human adenovirus B7 (HAdV-B7) might also be a cause of this disease.

HAdV-B7 is well recognized as a causative agent of neonatal disease and infections in immunocompromised patients. However, we identified an unusual cluster of 4 cases of severe ARD caused by this pathogen in immunocompetent adults in Dublin, Ireland. These patients had acute respiratory illness when they came to the emergency department of Mater Misericordiae University Hospital in Dublin. The patients came to the hospital over a 4-week period during the summer of 2017, and each patient required intensive care support for single-organ failure. Three patients required intubation and ventilation; all 4 patients recovered.

Three patients reported gastrointestinal and respiratory symptoms, as seen in Oregon, USA (*2*). Although co-infection with other viruses or bacteria has been described (*3*), only 1 patient in our cluster had a possible concomitant pathogen. None of the 4 patients were given antiviral therapy but all received antimicrobial drugs.

All 4 case-patients were either homeless or in temporary accommodations for homeless adults, but we did not identify any epidemiologic link. The Department of Public Health and temporary accommodation sites were notified to raise awareness and offer early testing of symptomatic persons. However, no additional cases were identified.

HAdV-B7 was identified by BLAST analysis (https://blast.ncbi.nlm.nih.gov/Blast.cgi) of viral hexon genes (*4*). Each virus had 100% identity within the region sequenced to a strain previously associated with respiratory illness in a military training camp in China (GenBank accession no. KP896481).

This cluster of HAdV-B7 causing severe ARD in immunocompetent adults appears to have no clear epidemiologic link. We agree that HAdV might be an underrecognized pathogen in severe community-onset ARD. Testing for viral respiratory pathogens should be considered in all patients and not just the immunocompromised.
